# Neuropathic Pain Masquerading as Dermatologic Symptoms: A Case of Misleading Cutaneous Presentation

**DOI:** 10.7759/cureus.64140

**Published:** 2024-07-09

**Authors:** Monica Khadka, Devaun M Reid, Erin McClure, Amanda Krenitsky, Catherine Kowalewski

**Affiliations:** 1 Internal Medicine, University of South Florida Morsani College of Medicine, Tampa, USA; 2 Dermatology, University of South Florida, Tampa, USA; 3 Dermatology, James A. Haley Veterans' Hospital, Tampa, USA

**Keywords:** lumbar, neuropathic, perineal pain, pudendal neuralgia, neuropathic pain

## Abstract

Neuropathic pain presenting as dermatologic symptoms can occur when damaged or dysfunctional nerves manifest with symptoms that resemble skin-related conditions. We present a case of a 62-year-old male who presented with burning pain and redness in the perineum and gluteal cleft. Initially, the patient was treated for dermatologic symptoms, resulting in the resolution of erythema. However, the pain persisted, prompting a neurologic workup. Despite the improvement of skin symptoms, the patient's pain persisted, prompting a neurological workup. Diagnostic imaging revealed significant degenerative changes in the lumbar spine, supporting a neuropathic etiology. This case highlights the importance of considering neurologic disorders in dermatologic practice, especially when cutaneous symptoms persist despite appropriate dermatological treatments.

## Introduction

Neuropathic pain can present with dermatologic symptoms such as erythema and irritation. This contrasts with the typical neuropathic sensations including numbness, tingling, and shooting pain [1]. This atypical presentation is not extensively detailed in medical literature and creates a diagnostic challenge. Patients with this condition often exhibit symptoms that mimic common dermatological disorders leading to potential misdiagnosis and delayed effective treatment. The case presented contributes to the literature by highlighting an example of this phenomenon, demonstrating the intricacies of its diagnosis and management. It underscores the need for a high degree of clinical suspicion for neuropathic explanations of dermatologic symptoms in patients at risk for peripheral neuropathies.

## Case presentation

A 62-year-old male with a past medical history of seborrheic dermatitis, coronary artery disease, myocardial infarction, cardiomyopathy, hypertension, type 2 diabetes mellitus, hyperlipidemia, and Charcot foot presented to the dermatology clinic with symptoms of pain and redness in the perineum and gluteal cleft for one month. On exam, an erythematous patch was found extending from the perineum through the gluteal cleft. The patient described the pain as stinging/burning with an intensity of eight out of ten. He is primarily mobility scooter-bound secondary to his comorbidities. 

The patient previously tried the following topical medications: lidocaine, triamcinolone 0.1%, desonide, clotrimazole and nystatin, ketoconazole, tacrolimus, clindamycin 1% solution, barrier creams vitamin A and D (A+D), and zinc. Trialed oral medications included fluconazole and amoxicillin. Non-pharmacological interventions included guidance on integumentary health self-care, lifestyle interventions with a donut pillow and foam mattress, and nutrition optimization. He declined patch testing. The initial differential diagnoses included gluteal dermatosis, candida intertrigo, seborrheic dermatitis, stage 1-2 pressure ulcers, perianal streptococcal infection, and anosacral amyloidosis. 

**Figure 1:**
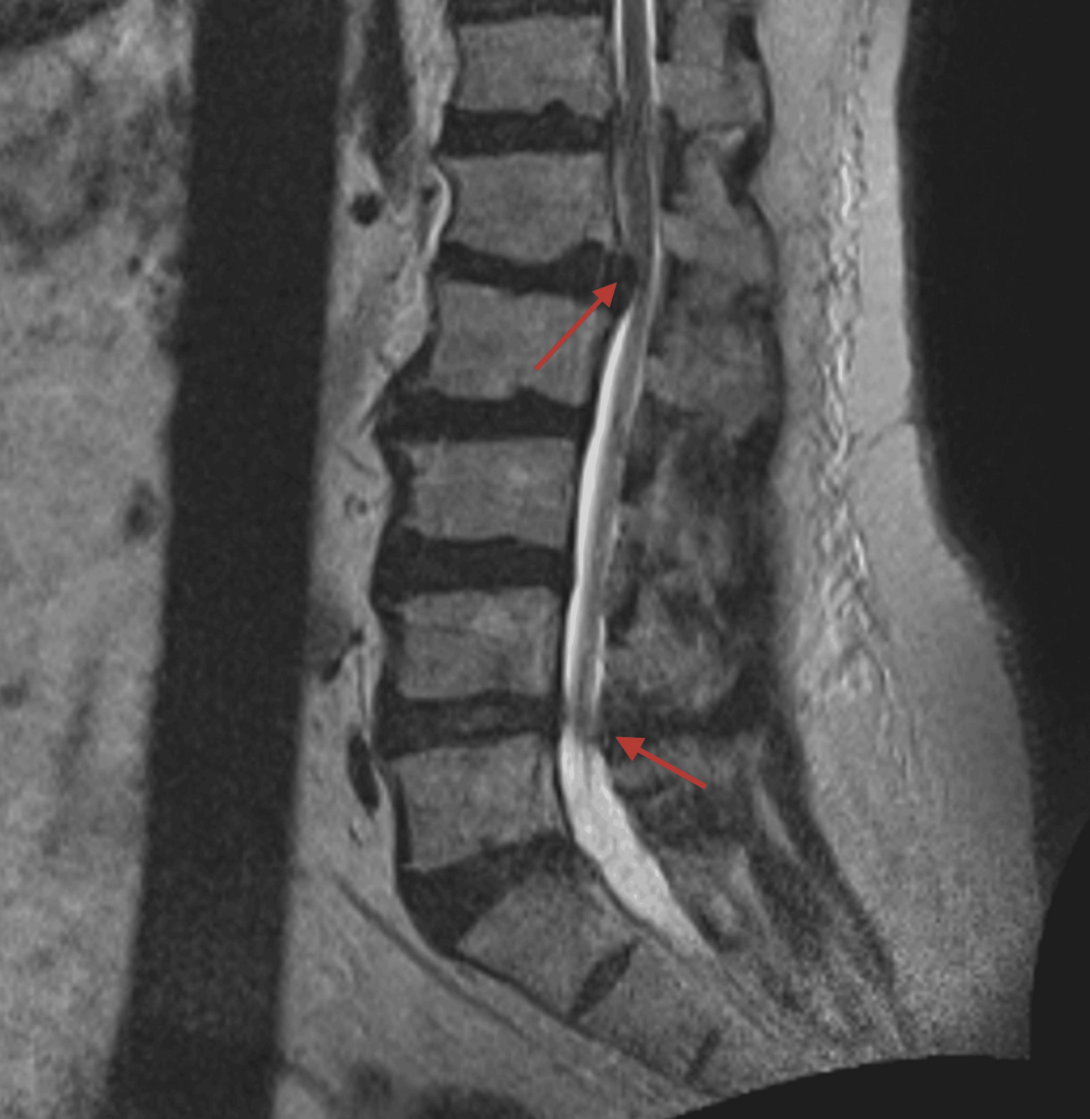
A T2-weighted sagittal MRI demonstrating multilevel degenerative disc disease with central disc protrusion, severe central spinal stenosis, and compression of the cauda equina nerve roots at L1-L2.

After a few months of treatment, he had improved skin findings but worsening pain with a burning quality. Thus, a dermatologic etiology was deemed less likely, and a neurologic etiology was considered. Additionally, a skin biopsy was deferred since the rash was resolved. Neurology and Neurosurgery were consulted and an MRI of the lumbar spine was ordered. Additionally, he was started on 300 mg of gabapentin nightly and referred to physical therapy. The MRI revealed several multilevel degenerative changes and structural abnormalities, such as spinal stenosis, neuroforaminal narrowing, and compression of the cauda equina nerve roots at L1-L2 (Figure 1). 

Per neurosurgery, these findings could be contributing to his perineal pain. While pudendal neuralgia was initially favored as the diagnosis, neurology’s final assessment did not support pudendal neuralgia as the patient lacked symptoms of saddle anesthesia and incontinence, and imaging did not capture S2-S4, which are involved in pudendal neuralgia. Instead, a pressure-related condition in the setting of type 2 diabetes mellitus and peripheral neuropathy was favored, with a treatment plan to increase gabapentin dosage and add methylprednisolone. Neurosurgery determined that immediate surgical intervention was not required, recommending medical optimization before considering surgery. Currently, his symptoms are improving on gabapentin, and the plan is to re-evaluate treatment after he completes physical therapy.

## Discussion

This patient initially presented with cutaneous symptoms, specifically, burning pain and redness in the perineum and gluteal cleft. This diagnosis was based on the visible cutaneous manifestations, aligning with the common clinical approach in dermatology where symptoms such as pain and erythema are attributed to skin-related etiologies [1-2]. While treatment with topical medications improved his cutaneous symptoms, his pain persisted, leading to the consideration of a neurological cause [3]. A neuropathic explanation of this patient’s symptoms was later supported by diagnostic imaging and symptomatic improvement with gabapentin. Neuropathic pain, often associated with conditions like diabetes mellitus and peripheral neuropathy, can manifest with atypical symptoms, including those that mimic dermatologic conditions [4].

The differential diagnosis of pudendal neuralgia was initially considered due to the location and nature of the patient’s symptoms [4-5]. However, the absence of saddle anesthesia and incontinence, along with the MRI findings that did not capture the typical pudendal neuralgia-affected areas (S2-S4), led to the exclusion of this diagnosis. Though Neurology decided this case is not well explained by pudendal neuralgia, it is possible that the MRI findings of this case do not correlate well to the affected area. Thus, a neurologic pathology is still present and the current treatment plan addresses this. Regardless of the etiology of this patient’s symptoms, pudendal neuralgia is important to consider as a differential diagnosis. Considering neuropathic causes in the differential diagnosis of dermatologic complaints can lead to earlier identification and optimized management of underlying neuropathic disorders [6-7]. Existing literature states that pudendal neuralgia often presents with non-specific symptoms, such as pain and numbness in the perineal area, that can easily be mistaken for other conditions and is frequently under/misdiagnosed [8]. Misdiagnosis of nerve entrapment syndromes often results in unnecessary pharmacological disease management when early, accurate identification of a neuropathic cause could have led to more effective treatment through physical therapy or surgery instead [9]. One limitation of this case was the patient's refusal of certain diagnostic assessments and interventions, like patch testing.

## Conclusions

This case highlights the importance of considering neuropathic explanations for dermatologic complaints, leading to earlier diagnosis and optimized management of neuropathic pain disorders. Clinicians should maintain a high level of suspicion for neurologic causes, even when standard dermatological treatments provide partial relief, to prevent misdiagnosis and unnecessary pharmacological treatment. While initial treatments targeting possible dermatologic etiologies provided partial relief in this case, the persistence of pain required further investigation into potential neuropathic causes. The exclusion of pudendal neuralgia, despite its symptomatic overlap, highlights the challenges in differential diagnosis when symptoms are atypical or non-specific. 
